# Supplementary material for: The Evolution and Characterization of the RNA Interference Pathways in Lophotrochozoa
Source: Genome Biol Evol. 2024 May 7;16(5):evae098. doi: 10.1093/gbe/evae098 (PMC11114477; doi:10.1093/gbe/evae098)
Supplement: evae098_Supplementary_Data [file evae098_supplementary_data.zip › Supplementary_materials.zip!/selected_miRNAs/NoGe_selected_miRNAs/known_miRNA_families_pdf_files/NMRB01000177.1_Notospermus_geniculatus_strain_Ushimado_scaffold177,_whole_genome_shotgun_sequence_6428.pdf]

Provisional ID	:	NMRB01000177.1_Notospermus_geniculatus_strain_Ushimado_scaffold177,_whole_genome_shotgun_sequence_6428
Score total	:	9801
Score for star read(s)	:	3.9
Score for read counts	:	9792.3
Score for mfe	:	1.7
Score for randfold	:	0
Score for cons. seed	:	3
Total read count	:	19219
Mature read count	:	19081
Loop read count	:	0
Star read count	:	138

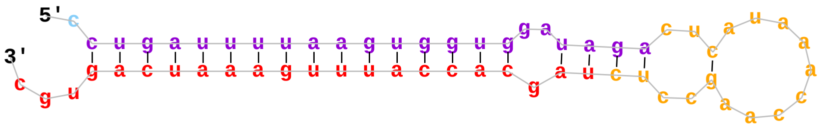

Genome coordinates: NMRB01000177.1 9797-9858

pre-miRNA sequence:
CUGAUUUUAAGUGGUGGAUAGACUGAUUUUAAGUGGUGGAUAGACUCAUAAACCAAGCCUCUAGCACCAUUUGAAAUCAGUGC

Mature sequence: UAGCACCAUUUGAAAUCAGUGC

Aligned mature sequence of the *N. geniculatus* pre-miRNA with the top 5 hits from the BLAST search against the pre-miRNA mirGeneDB set:

```
                            ********************
Nge_Mir-29-P1               uagcaccauuugaaaucagugc-
Ovu-Mir-29-P2_3p            uagcaccauuugaaaucaguuu-
Bge-Mir-29-P1k_3p           uagcaccauuugaaaucagugc-
Bge-Mir-29-P1l_3p           uagcaccauuugaaaucagugc-
Sko-Mir-29-P1_3p            uagcaccauuugaaaucaguguu
Pfl-Mir-29-P1_3p            uagcaccauuugaaaucagugu-
```
